# The British Medical Association Meeting at Portsmouth

**Published:** 1899-08-05

**Authors:** 


### THE BRITISH MEDICAL ASSOCIATION MEETING AT PORTSMOUTH.

The annual meeting of the British Medical Association was opened at Portsmouth on Tuesday. The Mayor and Corporation and the Council of the Association marched in procession to St. Mary's Church, where the sermon was preached by the Bishop of Winchester. At the same time high mass was celebrated at St. John's Roman Catholic Cathedral, where a sermon was preached by Mgr. Cahill.

The first general meeting was held in the afternoon, Dr. R. Saundby, president of the Council, opening the proceedings (in the absence, through indisposition, of Sir T. Grainger Stewart, president of the Association), after which the new president, Dr. J. Ward Cousins, took the chair.

The Mayor welcomed the Association to Portsmouth, waich was, he said, in one respect, the most important town in the kingdom, for it was the head-quarters of the British Navy.

When the Mayor and Corporation had left the room, the adoption of the annual report was moved by Dr. Saundby, who said that the revenue for the year amounted to £42,924. In seconding this Dr. Parsons, of Dover, mentioned that they had bought their premises in the Strand for £79,000, and that the profits on the year's work amounted to £4,700. Before the report was passed Mr. George Brown called attention to the attitude of the Privy Council in regard to the Midwives Bill put forward by the Midwives Association, which he described as a Bill which would be simply disastrous, and he moved as a rider that the Council of the Association be requested to make representations to the Lord President of the Council deprecating the adoption of Mr. J. B. Balfour's Bill as a Government measure. This was seconded by Dr. Brindley James, and much discussion followed of the usual animated character. In the end the rider was passed and the report agreed to.

After Dr. Muir had thanked the Association for the services it had rendered to the Scottish poor law medical officers, who, he said, were entirely at the mercy of the parish councils, from whom they had no right of appeal to the Local Government Board, Mr. Victor Horsley opened the "one-portal" question by moving, "That any draft Bill for the amendment of the Medical Acts shall contain, *inter alia*, provision for further direct representation of the profession on the General Medical Council, and for the establishment of a one-portal system of qualifying examination." He argued that, whatever might be done in regard to the primary and intermediate examinations, the final one should be alike everywhere.

After some discussion this was carried.

Then Mr. Brassey Brierley moved a resolution, deprecating the support given by Sir William Priestly in the House of Commons to the Midwives Bill, and this was carried.

Finally a resolution was passed condemning the conduct of the medical officers of the Coventry Provident Dispensary, a resolution which, however well deserved, seems to have within it the germ of a possible boycott and much heart burning. Altogether the proceedings served to demonstrate once more how critical the position of the Association might at any moment become if the resolutions of the general meetings carried with them any real force. If the Council chose to obey the behests of the general meetings on all the points raised, of course they have their policy laid down for them. We venture to opine that they will do nothing of the sort. Although we do not always agree with the way the Council manages affairs, we are quite clear that the safety of the Association depends on their sitting tight and keeping the reins in their hands. The Council, at the least, is representative of the branches past and present. The general meetings are representative of no one except the handful of members who sit them out.

In the evening the adjourned general meeting was held, when Dr. Ward Cousins delivered the presidential address.

On Wednesday the sectional meetings were commenced, and the address on medicine was delivered by Sir Richard Douglas Powell, Bart., M.D., F.R.C.P.

